# Segmental artery angioembolization as an efficient treatment modality for delayed hematuria with normal angiography: two case reports

**DOI:** 10.1186/s13256-024-04431-4

**Published:** 2024-03-12

**Authors:** Alireza Pakdel, Fardin Asgari, Razman Arabzadeh Bahri, Seyed Mohammad Kazem Aghamir

**Affiliations:** Urology Research Center, Tehran University of Medical Sciences, Tehran, Iran

**Keywords:** Nephrolithotomy, Percutaneous, Kidney Calculi, Angiography, Nephrectomy

## Abstract

**Background:**

Percutaneous nephrolithotomy is the most commonly used modality for the removal of kidney stones larger than 2 cm in size. Like other stone removal methods, percutaneous nephrolithotomy also has some complications, including bleeding and delayed hematuria. These complications are improved with conservative management and bed rest most of the time. However, it may require more invasive treatments. Angioembolization following an abnormal renal angiography is an efficient treatment modality for delayed hematuria. Furthermore, nephrectomy is suggested in uncontrolled cases of delayed hematuria when renal angiography is normal.

**Case presentation:**

We described two cases of uncontrolled delayed hematuria after percutaneous nephrolithotomy and angioembolization were carried out rather than potential nephrectomies. The first case was a 61-year-old Iranian man with left kidney stones, for whom percutaneous nephrolithotomy was planned. The patient was referred to the hospital after discharge with massive hematuria and had normal angiographic findings. An angioembolization was suggested for the patient and was carried out. His hematuria was dramatically improved within 30 minutes, and his hemoglobin level started to increase 2 days later. The second case was a 53-year-old Iranian man with kidney stones who was a candidate for right kidney percutaneous nephrolithotomy. The patient was referred to the hospital 4 days after discharge with a decreased hemoglobin level and massive hematuria. The patient had normal angiographic findings and was planned for angioembolization to control his hemorrhage, which dramatically decreased after the angioembolization within 60 minutes.

**Conclusion:**

Embolization of the segmental arteries of the targeted calyx can eliminate hematuria of the patient and prevent further nephrectomy.

## Introduction

Percutaneous nephrolithotomy (PCNL) is the first-line treatment for renal stones larger than 2 cm [1]. The efficacy and safety of PCNL have been approved in a variety of patient groups, including children, patients with obesity, and patients with renal anomalies [2]. Like other stone removal techniques, PCNL also has serious complications, including intraoperative hemorrhage or delayed hematuria. Delayed hematuria is an important adverse event following PCNL, which needs intensive and careful observation. In most cases, the mentioned complications will be improved with conservative management. However, more invasive techniques, such as angioembolization, may also needed as further treatment options [3–6]. Renal angiography is needed in cases with massive and continued hematuria, which can be followed by selective or over-selective angioembolization [7]. Herein, we report two cases with delayed hematuria following PCNL that were selected for angioembolization.

## Case presentation

This case report presents two cases on the basis of CARE guidelines. Written informed consent was obtained from the patients for publication of this case report and any accompanying images. A copy of the written consent is available for review by the editor-in-chief of this journal.

## Case 1

**Fig. 1:**
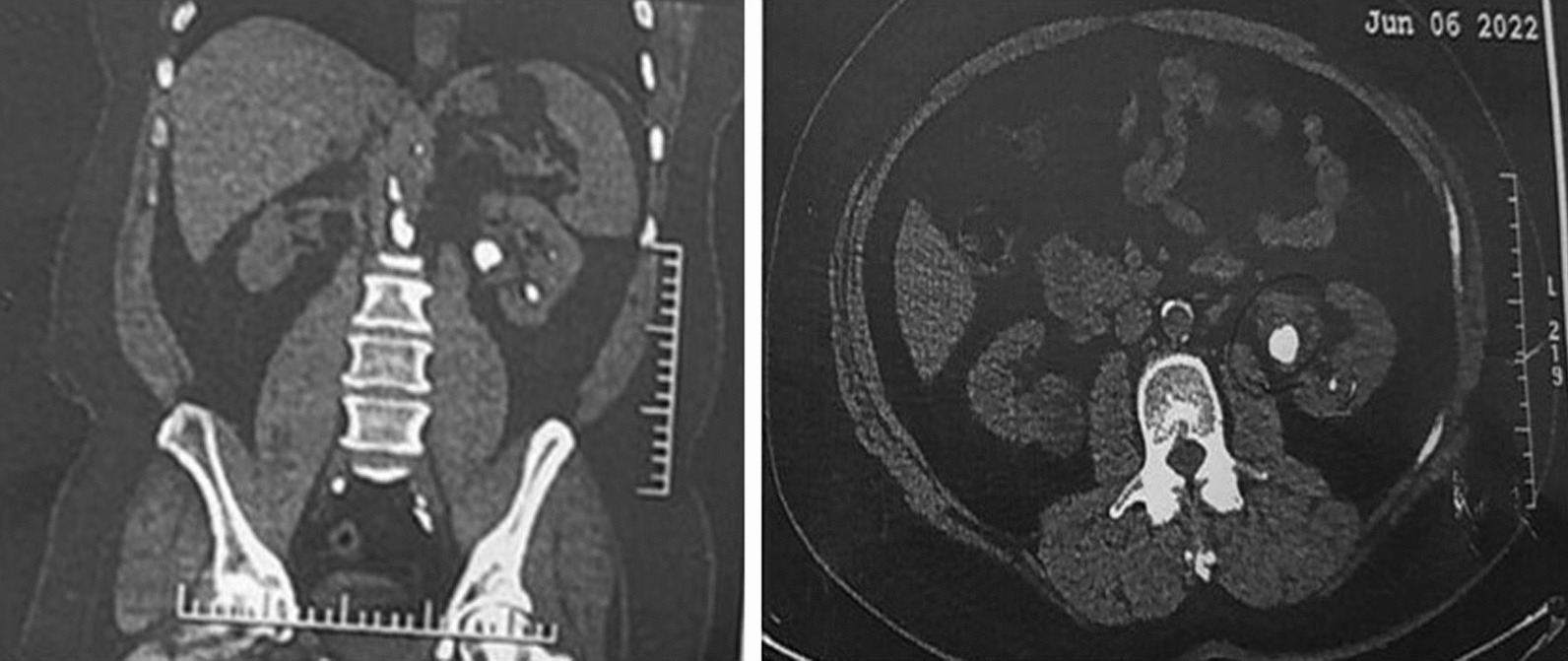
Computed tomography scan of the first case indicating urinary stones in the left kidney

A 61-year-old Iranian male was referred to the urology clinic in Sina Hospital, affiliated with the Tehran University of Medical Sciences, with left flank pain and microscopic hematuria. The patient denied any prior medical history and medication. A computed tomography (CT) scan was requested for the patient on the basis of examination and hematuria, which revealed a 22 × 15 mm stone in the middle calyx and a 7 × 7 mm stone in the lower calyx of the left kidney, and PCNL was thus planned for the patient (Fig. 1).

**Fig. 2:**
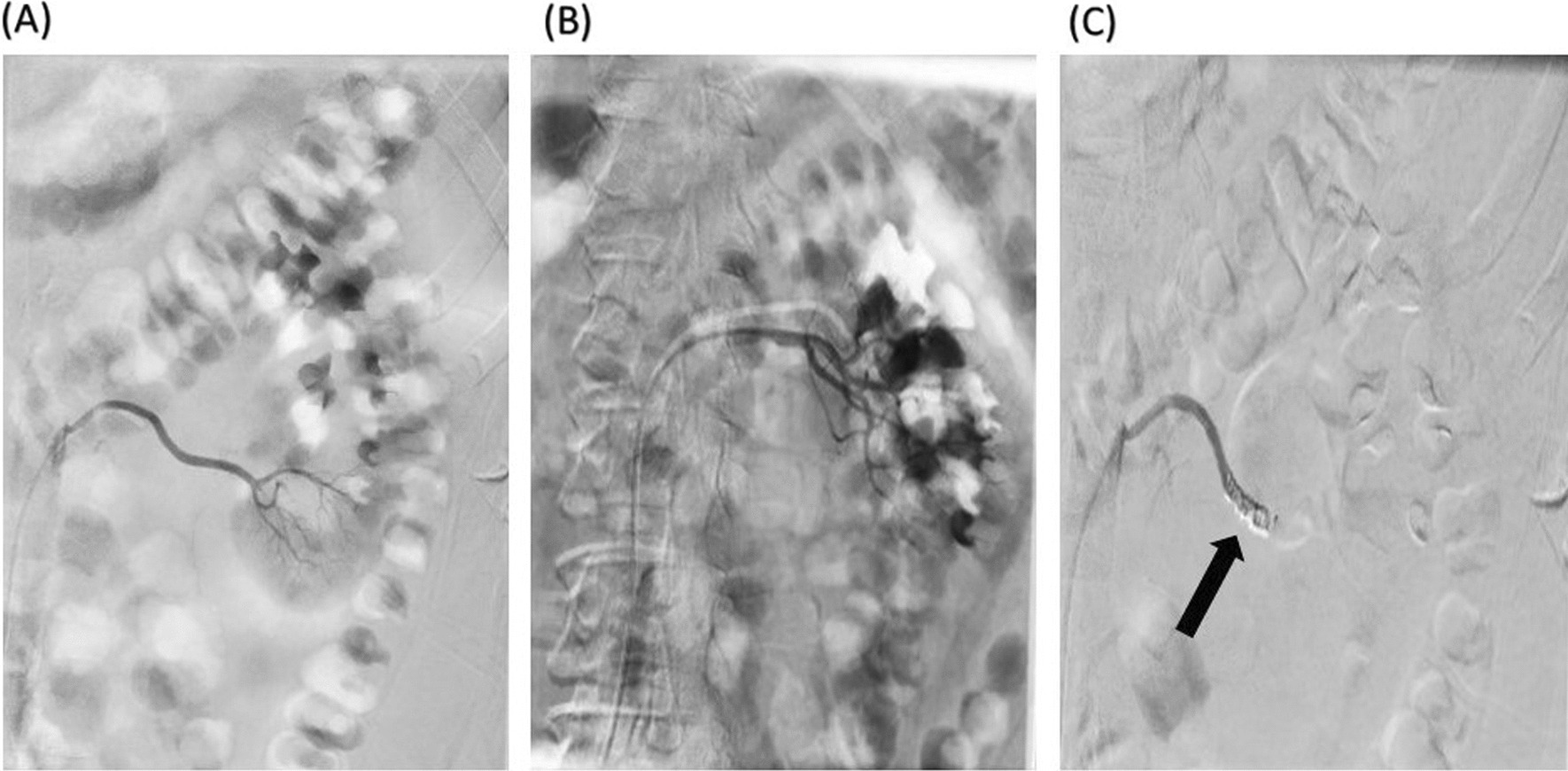
Left kidney arterial angioembolization of the first case. **A** lower calyx before coiling, **B** whole kidney before coiling, **C** after coiling in the lower calyx

Preoperative hemoglobin and creatinine levels of the patient were 13.8 g/dl and 1.1 mg/dl, respectively. General anesthesia was induced, and the patient was placed in lithotomy position. A 5 Fr catheter was inserted into the left ureter after using a needle. The patient was then placed in the prone position, and access was achieved to the posteroinferior calyx via fluoroscopy. After inserting the guidewire, the tract was dilated up to 28 Fr using an Amplatz dilator. The stones were then fragmented by pneumatic lithotripsy and removed using a nephroscope. The patient was discharged on the third postoperative day with a normal general condition. On the seventh postoperative day, the patient was referred to the hospital with massive hematuria and clots in the urine, which resulted in a decreased hemoglobin level of 6.5 g/dl. In addition, the patient had stable hemodynamic status (blood pressure: 110/60 mmHg; heart rate: 95 beats per minute). The transfusion of two units of packed red blood cells (pRBC) was performed for the patient over 2 days, and hemoglobin level increased to 9.5 g/dl. Hematuria continued, regardless of conservative management, and hemoglobin level decreased to 8 g/dl on the next postoperative day. Angioembolization was planned for the patient, and an angiography was performed, which did not reveal any vascular evidence as a source of hemorrhage, such as an arteriovenous fistula or vascular aneurysm. The angioembolization was thus not performed. The patient received a blood transfusion and was urged to complete bed rest. However, massive hematuria of the patient was still present, and hemoglobin level of the patient decreased to 7.5 g/dl within 48 hours. The patient was sent for angioembolization, and in the second attempt, a coil embolization of the segmental arteries was conducted (Fig. 2). Hematuria was dramatically improved after the second intervention within 30 minutes, and hemoglobin level of the patient started to increase 2 days later, up to 9 g/dl. The patient was discharged on the fifth postoperative day with a hemoglobin level of 10.3 g/dl with no signs of post-embolization syndrome, including flank pain or fever, and normal vital signs (blood pressure: 130/80 mmHg; heart rate: 82 beats per minute; temperature: 37.2 °C). The creatinine level of the patient was 1.43 mg/dl, which decreased to 1 mg/dl on follow-up evaluations. In terms of the amount of glomerular filtration rate (GFR), there was a slight decrease in the 6-month follow-up, which dropped from 98 ml/minute/1.73 m^2^ to 80 ml/minute/1.73m^2^.

## Case 2

**Fig. 3:**
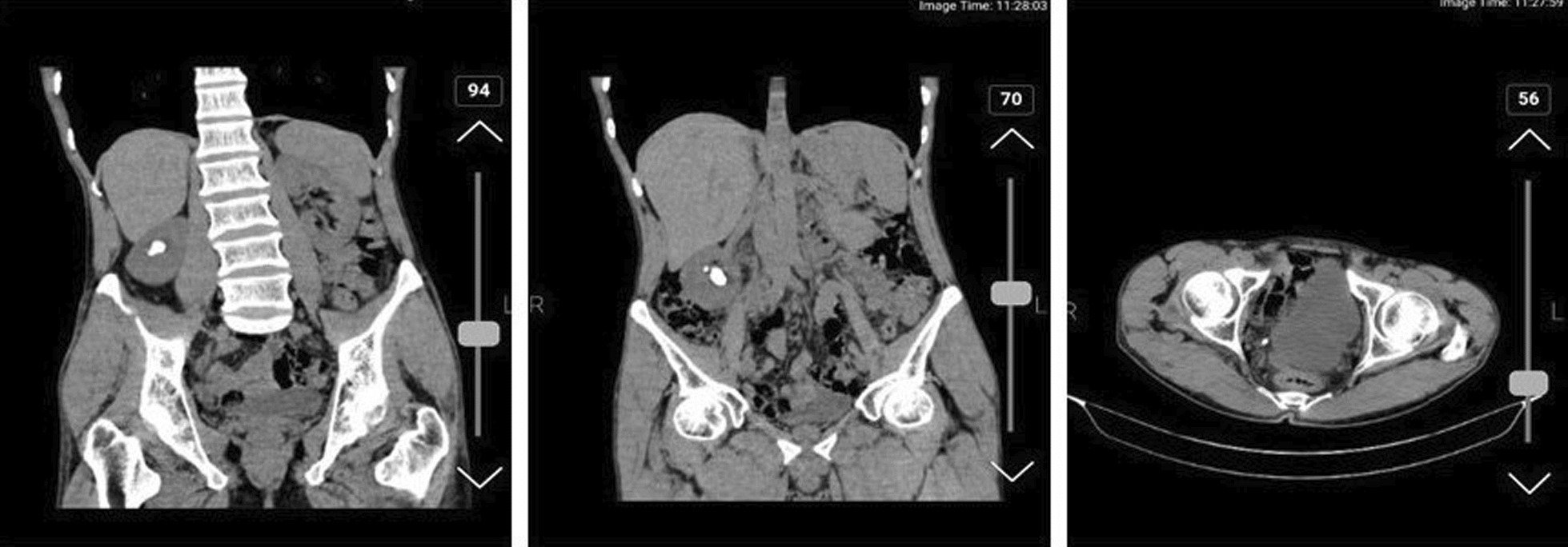
Computed tomography scan of the second case indicating urinary stones in the right kidney and ureter

A Iranian man in his 50s was referred to our clinic with right flank pain for 1 month. He denied any medical history or medications. In the physical examination, abdominal tenderness was negative. However, the costovertebral angle (CVA) tenderness was significant. In the urine analysis, hematuria was observed, and computed tomography (CT) scan revealed two stones in the middle and also the inferior part of the right kidney, with dimensions of 12 × 14 mm and 15 × 26 mm, respectively. In addition, a 5 × 6 mm stone in the inferior part of the right ureter was seen (Fig. 3).

**Fig. 4:**
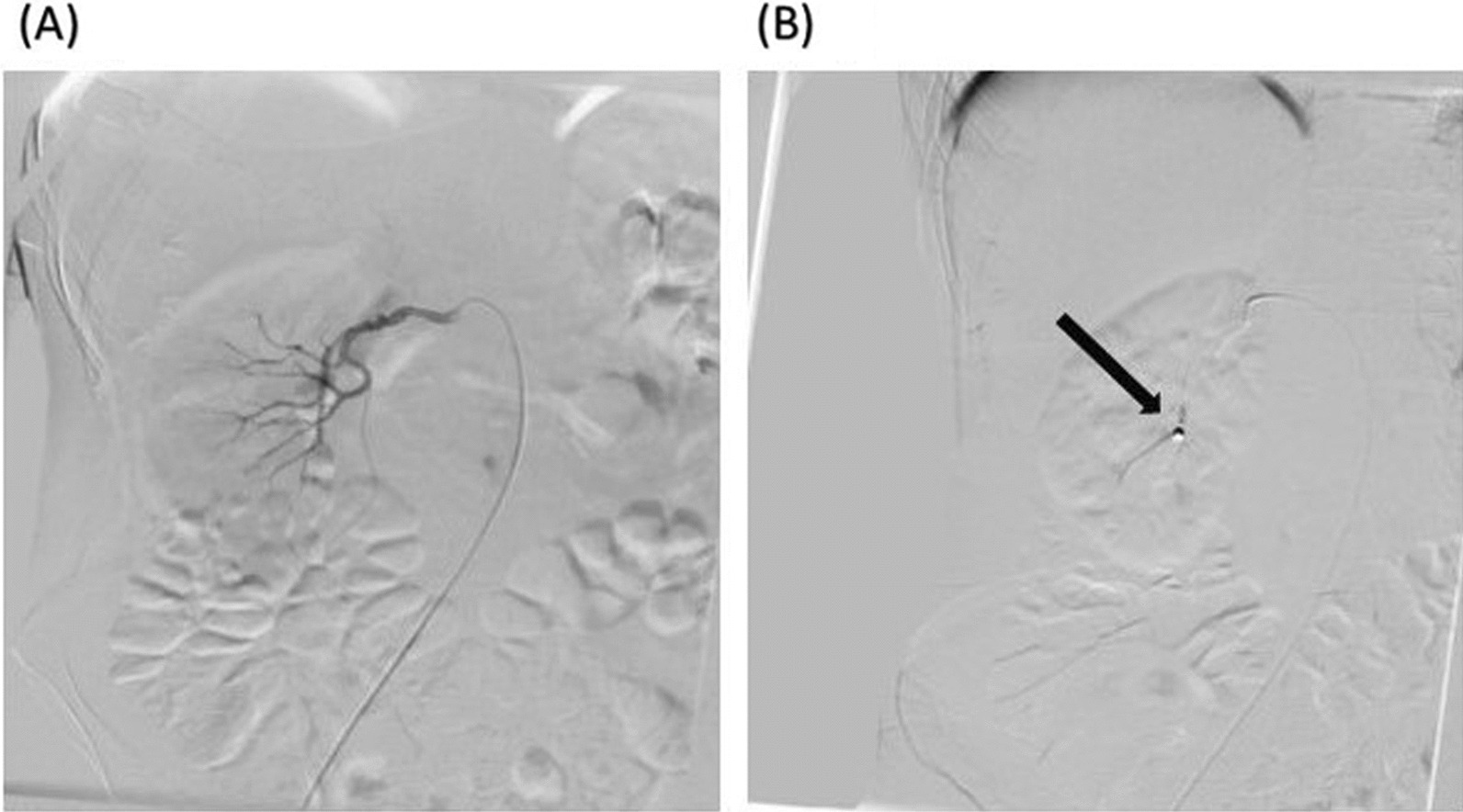
Right kidney arterial angioembolization of the second case. **A** lower calyx before coiling, **B** after coiling in the lower calyx (black arrow)

The patient was a candidate for right kidney PCNL. The preoperative hemoglobin and creatinine levels of the patient were 15.6 g/dl and 1.02 mg/dl, respectively. Prophylactic antibiotics were also given to the patient. Surgical procedures were similar to our first case. In addition, a double J stent was placed in the patient. On the second day of admission, the patient developed massive hematuria with a hemoglobin level of 10.8 g/dl, which decreased to 7.7 g/dl. The hemoglobin drop was improved by blood transfusion and conservative management, and the vital signs of the patient went back to normal ranges, with a blood pressure of 100/65 mmHg and a heart rate of 90 beats per minute. The patient was discharged from the hospital on the sixth postoperative day with a hemoglobin level of 10.1 g/dl and clear urine with no hematuria. The patient was referred to the hospital 4 days after discharge, which was 10 days after his surgery, with a decreased hemoglobin level of 9 g/dl and massive hematuria, which did not respond to conservative management and transfusion of two units of pRBC during 72 hours. Regardless of the blood transfusion, the hemoglobin level of the patient decreased to 8.6 g/dl, and he was suffering from gross hematuria. However, he had a stable hemodynamic status (blood pressure: 100/60 mmHg; heart rate: 97 beats per minute). The renal angiography of the patient was normal, and no arteriovenous fistula or vascular aneurysm was detected. Segmental angioembolization was planned to control hemorrhage. Coil embolization of the supplying artery of the lower calyx was performed on the basis of the prior attempt to remove the stones from the lower pole of the kidney during PCNL (Fig. 4). The hemorrhage dramatically decreased after the angioembolization, within 60 minutes, and the patient had stable hemodynamics and increased hemoglobin level within 48 hours after the intervention. The patient was discharged with a hemoglobin level of 10.9 g/dl, which increased to 12.4 g/dl in the evaluations. The GFR of the patient had a slight decrease from 112 ml/minute/1.73m^2^ to 96 ml/minute/1.73 m^2^, which was not significant.

## Discussion

In this study, we have presented two cases of delayed hematuria following PCNL, which underwent angioembolization of segmental arteries after normal angiography findings to control the hemorrhage and manage their hemoglobin drop. PCNL is the most commonly used modality for the removal of kidney stones larger than 2 cm in size, including complex and staghorn stones. Hemorrhage is one of the most significant complications of PCNL, which is mostly self-limiting. In 2–23% of PCNL cases, massive hemorrhage can occur, which requires blood transfusion [8–12]. Delayed hemorrhage, which is seen in 1–1.8% of PCNL cases, can originate from an arteriovenous fistula, a pseudoaneurysm, or trauma to the arteries or veins [13–18]. Venous bleeding can also occur in some patients, which is usually managed with conservative treatment using tamponade nephrostomy tubes [13]. A renal angiography can be helpful as a key factor when the vascular injury is highly suspicious. Additionally, hospitalization time can be reduced by shortening the gap between the hemorrhage and the intervention [19]. Angiography can be utilized as a first-line imaging modality [20]. Following the angiography, an angioembolization, with a success rate between 85% and 100%, is a kidney-sparing intervention [3, 5, 8, 9, 13, 21, 22]. In our study, the outcomes of angioembolization as an intervention for controlling the hemorrhage were positive, which was consistent with the results of prior studies. Regardless of the good and lifesaving results of angioembolization, it can result in some complications, including coil migration, renal artery dissection, loss of renal function, and non-target embolization [11]. It has been reported that angioembolization fails in 5% of cases in terms of stopping hemorrhage [11]. Angioembolization is performed when a pathologic defect can be seen. In addition, an angioembolization can be repeated after one prior unsuccessful attempt. Nephrectomy is considered a therapeutic option when the bleeding is not controlled after two episodes of angioembolization [9]. Considering a possible blind trauma to the tract in PCNL, which may not be seen in the angiography, performing a coil angioembolization of the artery of the targeted calyx may be extremely helpful as well as being a kidney-retaining intervention to prevent possible nephrectomy as a last-line therapeutic option. However, the justification for the embolization of an artery, despite the absence of discernible injury during angiography, is grounded in the acknowledgment of potential risks associated with the translocation of the nephroscope between distinct calyces. This is particularly pertinent in instances where stones are concurrently present in multiple calyces.

## Conclusion

It can be concluded that in cases with normal angiographic findings, embolization of the segmental arteries of the targeted calyx can eliminate hematuria of the patient and prevent further nephrectomy.

## Data Availability

Data will be provided on request.

## Abbreviations

CT: Computed tomography
CVA: Costovertebral angle
GFR: Glomerular filtration rate
PCNL: Percutaneous nephrolithotomy
pRBC: Packed red blood cells
